# The In Vitro Antibacterial Effects of Genipin Against Pathogens Commonly Associated With Infected Ulcerative Keratitis: A Canine Preliminary Study

**DOI:** 10.1167/iovs.67.3.18

**Published:** 2026-03-09

**Authors:** Inge J. M. Slenter, Annick A. G. Smit, Bas W. Schipper, Marian J. Broekhuizen-Stins, Linda van der Graaf-van Bloois, Robert P. L. Wisse, Sylvia C. Djajadiningrat-Laanen, Els M. Broens

**Affiliations:** 1 Department of Clinical Sciences, Surgery of Companion Animals, Veterinary Ophthalmology section, Faculty of Veterinary Medicine, Utrecht University, Utrecht, The Netherlands; 2 Faculty of Veterinary Medicine, Utrecht University, Utrecht, The Netherlands; 3 Department of Biomolecular Health Sciences, Veterinary Microbiological Diagnostic Centre, Faculty of Veterinary Medicine, Utrecht University, Utrecht, The Netherlands; 4 Xpert Clinics Oogzorg, Zeist, The Netherlands

**Keywords:** antimicrobial, cornea, antibiotic resistance, *gardenia jasminoides*, keratitis

## Abstract

**Purpose:**

To evaluate genipin as a novel antimicrobial therapeutic agent with established crosslinking abilities by investigating its antibacterial activity against canine ocular pathogens and exploring its pharmacodynamic profile.

**Methods:**

Nineteen bacterial strains were selected from frozen stock, including ten *Staphylococcus pseudintermedius* (five of which were methicillin-resistant [MRSP]), five *Pseudomonas aeruginosa*, and four *Streptococcus canis*. All strains were isolated from dogs with corneal stromal ulcerations. Minimum inhibitory concentration (MIC) and minimum bactericidal concentration (MBC) were examined; subsequently, time-kill assays were performed. Viability counts were performed at 0, 10, 30, 60, and 120 minutes.

**Results:**

Blue discoloration of the contents in the wells impeded the determination of the MIC values for all pathogens. MBC values ranged from 0.1% for MRSP, 0.075%–0.125% for *S*. *pseudintermedius* and *P*. *aeruginosa*, and 0.25%–0.5% for *S. canis.* Time-kill analysis demonstrated a mean bacteriostatic effect of genipin against staphylococci at 1 × MBC after 120 minutes, and a mean bactericidal effect after 120 minutes at 2.5 × MBC and after 60 minutes at 5 × MBC. No antibacterial effect was measured for *P*. *aeruginosa* within the tested timeframe and genipin concentrations.

**Conclusions:**

Genipin exhibited promising antibacterial activity against all tested isolates, including MRSP. We suggest that genipin acts as a concentration-dependent antimicrobial agent, demonstrating bactericidal activity at higher concentrations against staphylococci. *P*. *aeruginosa* appeared less susceptible to the short-term effects of genipin. Further investigation is warranted to elucidate genipin's full antimicrobial potential and clinical performance.

Infected ulcerative keratitis is a common ocular disease associated with high ocular morbidity in both humans and animals, where treatment becomes particularly challenging when (multi)drug-resistant (MDR) bacteria are involved.^1^^,^^2^ The potential consequences for the eye can be severe, including keratomalacia, corneal perforation, scarring, loss of vision, or even loss of the eye.^3^^–^^6^ Bacteria commonly associated with infected ulcerative keratitis in humans and dogs include *Staphylococcus* species, *Pseudomonas aeruginosa*, and *Streptococcus* species.^7^^–^^13^ These bacteria have been shown to regularly exhibit MDR (resistance to ≥3 antibiotic classes) in several recent veterinary studies,^7^^–^^9^^,^^12^ two of which reported an increase in MDR over time.^8^^,^^12^ The prevalence of MDR bacteria ranged from 20% to up to 52.5% of cultured isolates,^7^^,^^9^ which is comparable to the numbers reported in human ophthalmology.^14^^,^^15^ This high prevalence of MDR bacteria highlights a significant “One Health” issue, emphasizing the need to explore alternative antimicrobial compounds and adjunctive treatment strategies to combat these corneal infections.

One potential alternative antimicrobial compound is genipin, a naturally occurring, non-glycosidic iridoid. It is the active metabolite derived from geniposide, which is extracted from the *Gardenia jasminoides* plant.^16^ In East Asia, genipin is commonly used as a natural food coloring additive because it reacts with primary amino acids to produce a blue pigment.^17^^,^^18^ Genipin and its derived blue pigments have sparked interest in several fields of medicine because of their wide range of activities, including anti-inflammatory^19^^–^^21^ and antioxidative effects.^22^^,^^23^ Additionally, genipin demonstrates noteworthy antimicrobial activity, with reported efficacy against both Gram-positive and Gram-negative bacterial strains.^24^^–^^28^ Koudouna et al.^26^ reported a bactericidal effect of genipin against corneal pathogens, including *Staphylococcus aureus* and *P.* *aeruginosa*. Furthermore, genipin treatment reduced bacterial load and suppressed neutrophil infiltration, alleviating the severity of bacterial keratitis in an in vivo study in rabbits.^28^ Interestingly, genipin exhibited antimicrobial activity against MDR *Escherichia coli*, and a synergistic effect was observed when genipin was combined with norfloxacin.^27^

In addition to its promising antimicrobial properties, genipin has been shown to increase extracellular matrix synthesis, preventing corneal perforation.^29^ Genipin activates keratocytes, and, based on the presence of highly reflective structures observed with in vivo confocal microscopy, it may additionally promote collagen fiber secretion.^30^ Furthermore, genipin-treated biological tissues—including corneal tissue—exhibit increased resistance to enzymatic digestion.^31^^–^^34^ Together, these effects may prove valuable in the multimodal treatment of infected ulcerative keratitis. As previously suggested,^26^^,^^28^ genipin likely exerts a multifactorial effect related to its crosslinking abilities.^31^^,^^33^^,^^35^^–^^40^ However, the precise mechanism of action of genipin has not yet been elucidated.

A time–kill assay can provide empirical data to further explore how drug concentrations relate to their effects on bacteria. To date, no time–kill studies have been conducted to evaluate the activity of genipin against ocular pathogens. Further investigating the pharmacodynamic and pharmacokinetic properties of genipin is an essential next step toward providing clinically relevant insights for ophthalmic applications. The objective of the current study was to assess whether genipin has in vitro antibacterial activity against pathogens commonly found in canine corneal stromal ulcerations and to explore its pharmacodynamic profile.

## Methods

### Bacterial Strains

To reflect clinically relevant ocular pathogens, 10 *Staphylococcus pseudintermedius* (including five methicillin-resistant (MRSP)), five *P.* *aeruginosa,* and four *Streptococcus canis* strains, stored at −80°C, were selected from the collection of the Veterinary Microbiological Diagnostic Center of Utrecht University, The Netherlands. All strains originated from samples from canine patients with corneal stromal ulcerations, submitted by the ophthalmology staff of the University Clinic for Companion Animals, Utrecht University, The Netherlands, for diagnostic evaluation. Bacteria were grown aerobically on blood agar plates (Columbia Agar with Sheep Blood) for 18–22 hours at 37°C. *S.* *canis* species were grown under microaerophilic conditions (12% O_2_, 5% CO_2_, 3.8% H_2_) in vacuum-sealed Anoxomat jars (Nova Biomedical, Waltham, MA, USA.)

### Feasibility Study

A pilot study was conducted to test the feasibility of the experimental procedures and determine the final concentration range of genipin to be tested. The highest genipin concentration achievable with minimal dimethyl sulfoxide (DMSO) content was evaluated. One strain of each isolate was included. Because streptococci are fastidious facultative anaerobes, *S. canis* were cultured under aerobic and under microaerophilic conditions to evaluate the impact of oxygen availability on their response to genipin.

### Inhibitors

Genipin (98% purity, high precision liquid chromatography [HPLC]) was purchased from Challenge Bioproducts (Douliu, Taiwan). Genipin exhibits high solubility in organic solvents such as DMSO (≥25–50 mg/mL), but limited aqueous solubility.^41^ Therefore a DMSO–PBS mixture was used to prepare the stock solutions. Stock solution concentrations could not be achieved in PBS because of precipitation on dilution. An initial stock solution of 25 mg/mL genipin (in 25% DMSO) was prepared by dissolving 200 mg of genipin in 2 mL of 100% DMSO (Sigma-Aldrich Corp., St. Louis, MO, USA), followed by dilution with 6 mL of PBS. A second stock solution of 10 mg/mL genipin (10% DMSO) was prepared by further diluting 5 mL of the first stock solution with an additional 7.5 mL of PBS. Fresh stock solutions of genipin were prepared at the start of each experiment.

Because of reported antibacterial effects of DMSO, one strain of each isolate was tested against 2%, 2.5%, and 5% DMSO. Two strains of *S. canis* were additionally tested against 7.5% and 10% DMSO. To avoid unnecessary expenditures, DMSO concentrations below 2% were not tested.

### Determination of Minimum Inhibitory Concentration and Minimum Bactericidal Concentration

The minimum inhibitory concentration (MIC) and minimum bactericidal concentration (MBC) of genipin were determined using the broth microdilution method, according to the Clinical and Laboratory Standards Institute (CLSI) guidelines.^42^ Suspensions in normal saline were prepared from all strains to obtain an optical density of 0.5 McFarland. Hereafter, the suspensions were further diluted in cation-adjusted Mueller-Hinton broth (BD BBL [Becton Dickinson, Franklin Lakes, NJ, USA]; CAMHB [Thermo Fisher Scientific, Waltham, MA, USA]) to achieve a bacterial concentration of approximately 2 × 10^6^ CFU/mL. Strains of *S*. *canis* were diluted in a separate CAMHB with 10% lysed horse blood.

For each experiment, three 96-well microtiter plates were used. Each well was filled with a total volume of 200 µL, consisting of 50 µL of inoculum (final concentration of approximately 5 × 10^5^ CFU/mL) and varying volumes of nuclease-free molecular-grade water and genipin solution. Eight genipin/DMSO concentrations (0.025%/0.25%, 0.05%/0.5%, 0.075%/0.75%, 0.1%/1%, 0.125%/1.25%, 0.2%/2%, 0.25%/2.5%, and 0.5%/5%) were tested against MRSP, *P*. *aeruginosa,* and *S*. *pseudintermedius* strains using the 10 mg/mL stock solution. Based on our pilot study results, a different range of concentrations (genipin/DMSO; 0.2%/2%, 0.25%/2.5%, 0.5%/5%, 0.75%/7.5%) was used for the *S*. *canis* strains, prepared from a 25 mg/mL stock solution, and was incubated at 37°C for 22 hours. Staphylococci and *P. aeruginosa* strains were grown under aerobic conditions, streptococci under microaerophilic conditions. The MIC was defined as the lowest genipin concentration that inhibited bacterial growth, as assessed by both visual inspection (turbidity of the contents in the well) and spectrophotometry.

To determine the MBC values, the track dilution method was adapted as previously described by Jett et al.^43^ with some minor modifications. After the 22-hour incubation period, 10 µL aliquots of bacterial suspension were cultured in three to four rows on 90 mm round blood agar plates. The plates were incubated at 37°C for 20 hours. The MBC was defined as the minimum concentration of genipin that reduced the initial microbial population to <5 colonies, representing a 3-log reduction.^42^ All tests included growth and sterility controls and were performed in triplicate on three separate occasions.

### Broth Types

In an attempt to circumvent the blue discoloration of genipin, two types of broth, CAMHB and Luria-Bertani broth (LBB; Xebios Diagnostics GmbH, Dusseldorf, Germany), were compared during the pilot study. Sterile 0.85% saline solution was used as a negative control. Additionally, the time until blue discoloration was reported at 0, 5, 10, 30, 60, and 120 minutes.

### Time-Kill Assay

After the MBC determination, a time-kill assay was performed with one strain each of *S*. *pseudintermedius*, MRSP, and *P*. *aeruginosa* (see Table 1)*.* The method used in this study was adapted from previously published data.^44^^–^^46^ Bacterial colonies from the overnight blood agar plate were transferred into glass test tubes containing 12 mL Mueller-Hinton II Broth (MHB; AnalytiChem Netherlands B.V., Mijdrecht, The Netherlands) and incubated for 16 hours at 37°C. The optical density (OD) at 600 nm of the overnight culture in MHB was measured using the Ultrospec 10 cell density meter. A sample of this culture was then diluted to an OD of 0.02 in a total volume of 4 mL MHB. The MHB vials containing the inoculum were subsequently placed on an orbital shaker (200 rotations per minute; rpm) in the incubator at 37°C. Optical density was measured every hour until an OD of ≥0.3 was reached. Then, every 15 minutes until an OD between 0.4 and 0.6 was reached (verification of bacteria in the logarithmic phase), after which 150 µl of the inoculum was added to prewarmed (37°C) and prefilled 96-well microtiter plates. The time required to achieve the logarithmic growth phase was recorded in minutes. Three wells were prefilled with genipin concentrations of 1×, 2.5×, and 5× the MBC for each respective strain, together with the inoculum; the total volume of the wells contained 300 µl, which constituted equivalent amounts of inoculum and genipin. The plates were placed on an orbital shaker (200 rpm) and incubated at 37°C. Viability counts were performed at 0, 10, 30, 60, and 120 minutes by plating 10-fold serial dilutions of 10 µL aliquots using the previously described track dilution method. For each concentration and time point, colonies were counted from the first dilution yielding a countable range of three to 30 colonies, and the CFU/mL was subsequently calculated. Bacteriostatic activity was defined as a ≥1 log reduction, and bactericidal activity as a ≥3 log decrease in CFU/mL compared to the bacterial concentration of the growth control. All tests included growth and sterility controls and were performed in triplicate on three separate occasions.

**Table 1.** MBC for Genipin Against 19 Clinical Strains of Bacteria Originating From Samples From Canine Patients With Corneal Stromal Ulcerations

Bacterial Isolates	Internal Reference Number	Environment	MBC
*S. pseudintermedius*	2110209036	Aerobic	0.125%
*S. pseudintermedius*	214062605101-1	Aerobic	0.125%
*S. pseudintermedius*	221070607401-3	Aerobic	0.125%
*S. pseudintermedius* ^*^	222052006101-1	Aerobic	0.1%
*S. pseudintermedius*	222083104801-1	Aerobic	0.075%
MRSP^*^	2130426050	Aerobic	0.1%
MRSP	217030205401-1	Aerobic	0.1%
MRSP	217110905301-1	Aerobic	0.1%
MRSP	217121404701-1	Aerobic	0.1%
MRSP	223022007801	Aerobic	0.1%
*P. aeruginosa*	113420	Aerobic	0.075%
*P. aeruginosa* ^*^	9406936	Aerobic	0.1%
*P. aeruginosa*	8306	Aerobic	0.1%
*P. aeruginosa*	213111101101-1	Aerobic	0.1%
*P. aeruginosa*	221090302201-1	Aerobic	0.1%
*S. canis*	217010902001-2	Microaerophilic	0.5%
*S. canis*	221111806401-1	Microaerophilic	0.5%
*S. canis*	222030302901-2	Microaerophilic	0.5%
*S. canis*	222122702801-2	Microaerophilic	0.5%

* The strains that were used for the time-kill assay.

*S.* *canis* did not enter the logarithmic growth phase under aerobic conditions. Although the study set-up was attempted under microaerophilic conditions, delays in gas exchange, intermittent aerobic exposure, and time outside the incubator likely hindered bacterial proliferation. Therefore *S. canis* is not included in the time-kill assay.

### Statistical Analysis

Data are presented descriptively. The mode of all three replicates was used to determine the MBC. Time-kill analysis data are presented as mean values ± standard deviations. The statistical software R, version 4.5.0, was used to analyze the data and construct line graphs.

## Results

### MIC and MBC

After 20 hours of incubation, all tested genipin concentrations resulted in dark blue discoloration of the well contents, hampering visual inspection of bacterial growth (Supplementary Fig. S1.1). Spectrophotometry results were similarly indistinguishable from each other (Supplementary Fig. S1.2).

The MBC values for genipin against the 19 tested clinical strains are shown in Table 1. All tested strains appeared susceptible to genipin within the tested concentration range. *S. canis* appeared less susceptible to genipin when incubated in a microaerophilic environment compared to aerobic conditions (0.1% vs. 0.5%; Supplementary Table S1.1). Evaluation of bacterial growth in three or four (“tracks”) was feasible using 90 mm round agar plates (Supplementary Fig. S1.3). DMSO concentrations up to 7.5% had no antibacterial effect (Supplementary Table S1.2)

### Broth Types

Blue discoloration appeared within 5 minutes, varying with genipin concentration and broth type (Supplementary Fig. S1.4). After 20 hours at 37°C, all broth types and genipin concentrations exhibited similar dark discoloration. No blue discoloration was observed in the 0.85% saline solution group.

### Time-Kill Assay

The time required to reach the logarithmic growth phase ranged from 215 to 255 minutes for both MRSP and *S*. *pseudintermedius and* 285 to 360 minutes for *P. aeruginosa*.

**Figure 1.**
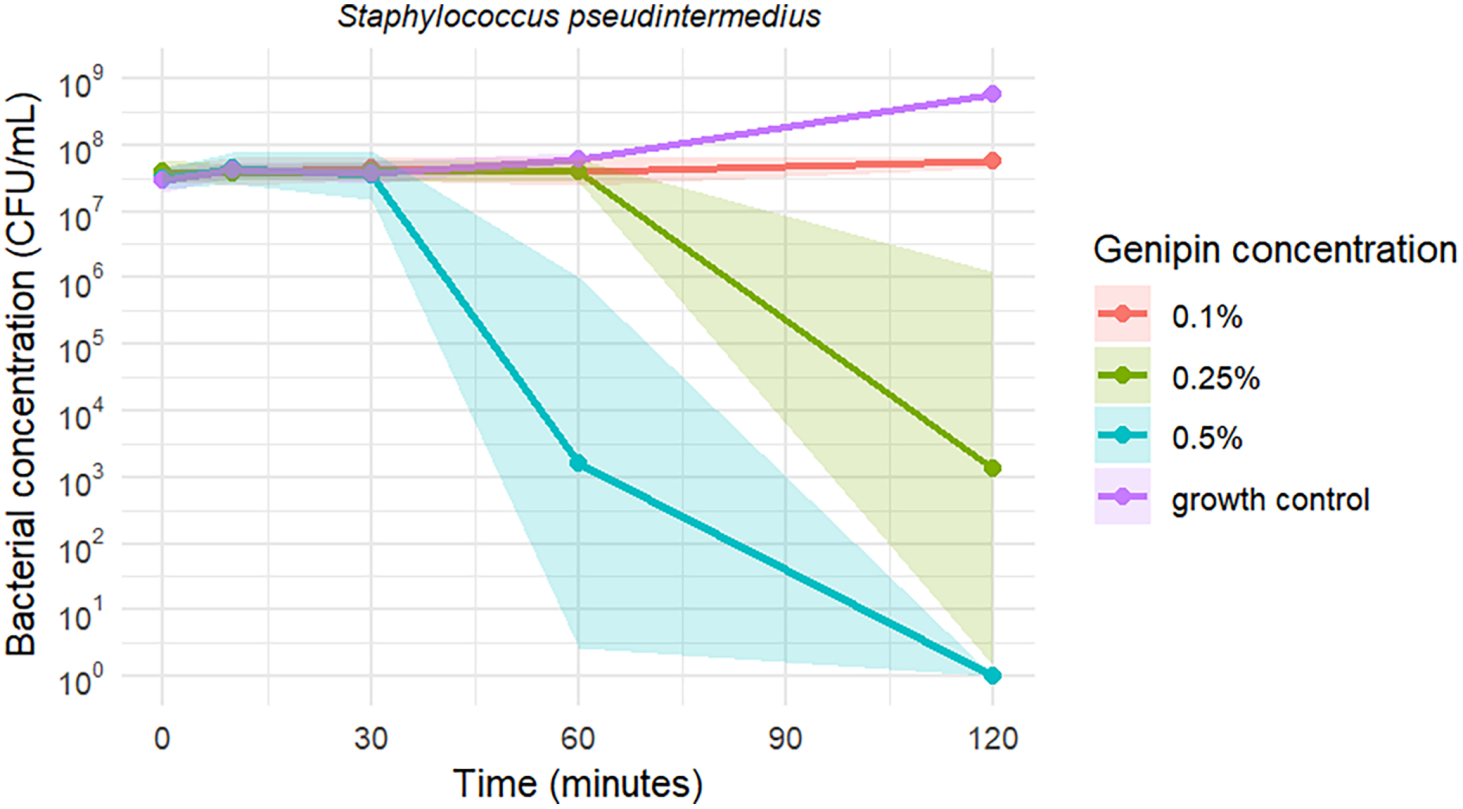
Time-kill curves of S. *pseudintermedius* against three genipin concentrations, including a growth control. Data represent mean values ± SD. Note the logarithmic scale along the y-axis.

**Figure 2.**
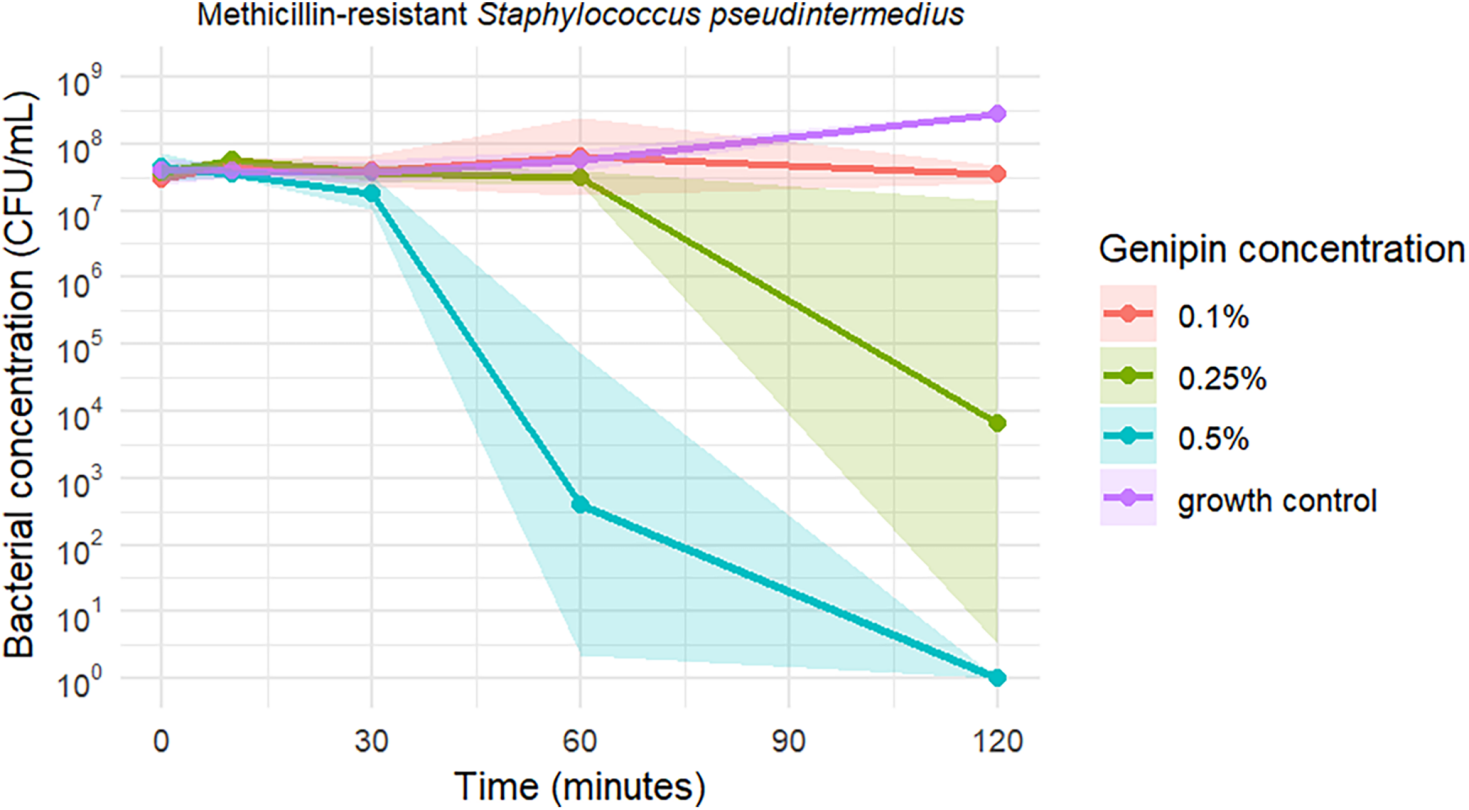
Time-kill curves of MRSP against three genipin concentrations, including a growth control. Data represent mean values ± SD. Note the logarithmic scale along the y-axis.

**Figure 3.**
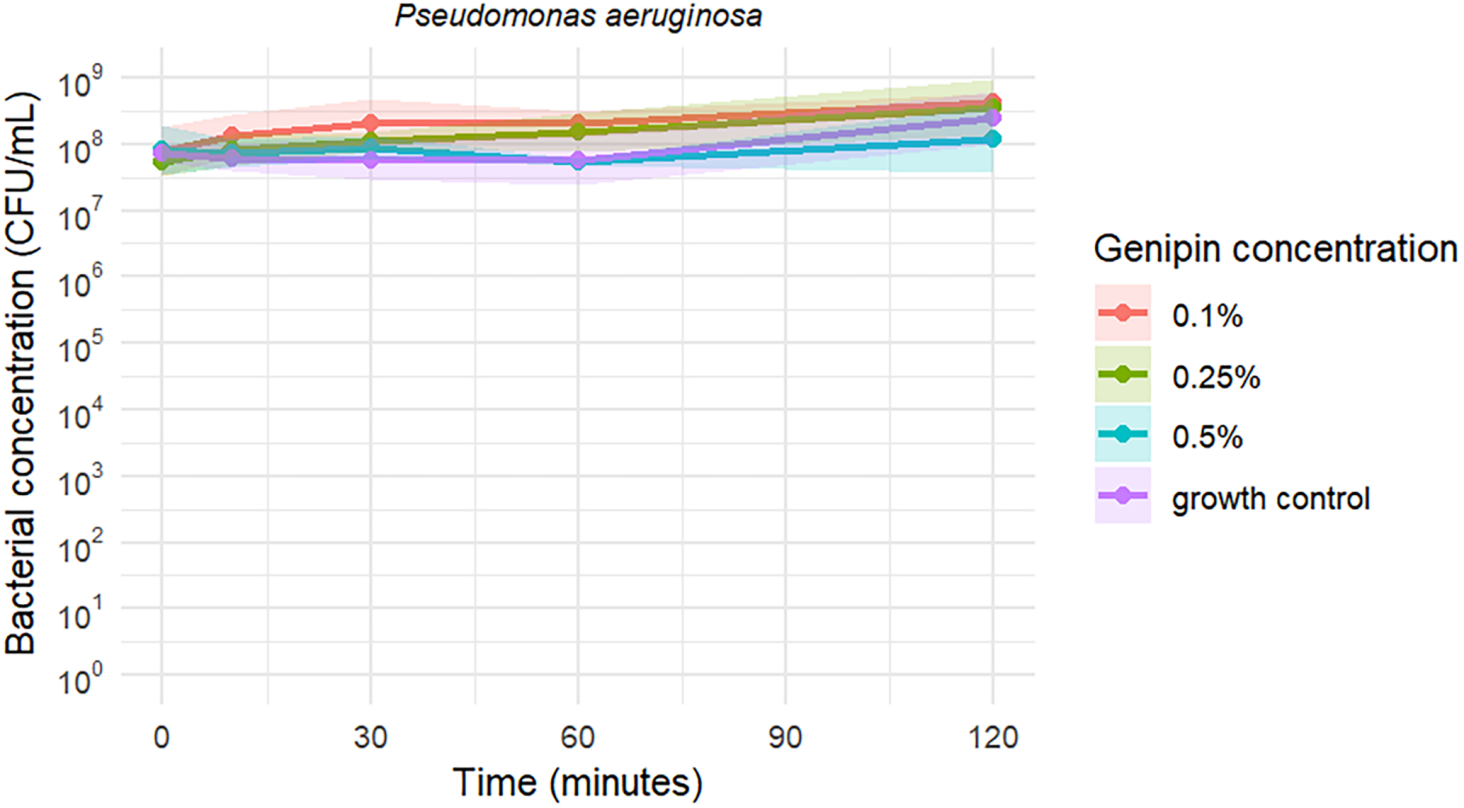
Time-kill curves of *P. aeruginosa* against three genipin concentrations, including a growth control. Data represent mean values ± SD. Note the logarithmic scale along the y-axis.

Time-kill curves of *S*. *pseudintermedius*, MRSP, and *P. aeruginosa* exposed to the three tested genipin concentrations are shown in Figures 1, 2, and 3, respectively. Genipin 0.1% resulted in a mean bacteriostatic effect for staphylococci after 120 minutes of incubation. Genipin 0.25% (2.5 × MBC) and 0.5% (5 × MBC) induced a mean bactericidal effect against the tested staphylococci after 120 and 60 minutes, respectively. As illustrated in Figures 1, 2, and 3, the standard deviation of the measurements is notably high at 60 minutes for the 0.5% genipin concentration, and at 120 minutes for the 0.25% concentration in both staphylococci species. Table 2 presents the CFU/mL values recorded at each time point during the time-kill assays. No bacterial killing was achieved for *P. aeruginosa* within the tested time frame and genipin concentrations.

**Table 2.** Time-Kill Analysis Results in CFU/mL Per Bacterial Isolate, Day, Time Point, and GP Concentration

	EXP 1				EXP 2				EXP 3			
	GP 0.1%	GP 0.25%	GP 0.5%	GC	GP 0.1%	GP 0.25%	GP 0.5%	GC	GP 0.1%	GP 0.25%	GP 0.5%	GC
*S. pseudintermedius*												
0 minutes	4.5 × 10^7^	4 × 10^7^	3.8 × 10^7^	4 × 10^7^	4 × 10^7^	2.5 × 10^7^	2 × 10^7^	1.9 × 10^7^	3 × 10^7^	6 × 10^7^	4 × 10^7^	3 × 10^7^
10 minutes	7 × 10^7^	5 × 10^7^	7 × 10^7^	4.5 × 10^7^	3 × 10^7^	3.7 × 10^7^	2.4 × 10^7^	3.2 × 10^7^	3 × 10^7^	3 × 10^2^	5 × 10^7^	5 × 10^7^
30 minutes	4 × 10^7^	2.7 × 10^7^	1.7 × 10^7^	3 × 10^7^	3 × 10^7^	5 × 10^7^	9 × 10^7^	6 × 10^7^	7 × 10^7^	5 × 10^7^	2.7 × 10^7^	3 × 10^7^
60 minutes	3 × 10^7^	4 × 10^7^	0^†^	5 × 10^7^^‡^	7 × 10^7^	2.8 × 10^7^	1 × 10^5^^*^	8 × 10^7^^‡^	3 × 10^7^	6 × 10^7^	4.5 × 10^4^^†^	6 × 10^7^^‡^
120 minutes	7 × 10^7^	0^†^	0^†^	3 × 10^9^^‡^	5 × 10^7^	3 × 10^3^^†^	0^†^	3 × 10^8^^‡^	5 × 10^7^	8 × 10^5^^*^	0^†^	2 × 10^8^^‡^
MRSP												
0 minutes	3 × 10^7^	3.3 × 10^7^	6 × 10^7^	2.4 × 10^7^	2.8 × 10^7^	5 × 10^7^	2.5 × 10^7^	6 × 10^7^	3 × 10^7^	3 × 10^7^	6 × 10^7^	4 × 10^7^
10 minutes	5 × 10^7^	6 × 10^7^	3 × 10^7^	3 × 10^7^	4 × 10^7^	6 × 10^7^	3.7 × 10^7^	5 × 10^7^	6 × 10^7^	5 × 10^7^	4 × 10^7^	4 × 10^7^
30 minutes	2.1 × 10^7^	4 × 10^7^	1 × 10^7^	3 × 10^7^	5 × 10^7^	2.7 × 10^7^	2.1 × 10^7^	3 × 10^7^	6 × 10^7^	5 × 10^7^	3 × 10^7^	6 × 10^7^
60 minutes	2.6 × 10^7^	2.6 × 10^7^	0^†^	4 × 10^7^^‡^	3.6 × 10^7^	4 × 10^7^	5 × 10^3^^†^	8 × 10^7^^‡^	3 × 10^8^	2.8 × 10^7^	1.3 × 10^4^^†^	6 × 10^7^^‡^
120 minutes	3 × 10^7^	0^†^	0^†^	3 × 10^8^^‡^	2.8 × 10^7^^*^	1 × 10^6^^*^	0^†^	3 × 10^8^^‡^	5 × 10^7^	3 × 10^5^^*^	0^†^	2.6 × 10^8^^‡^
*P. aeruginosa*												
0 minutes	3 × 10^7^	3 × 10^7^	3.1 × 10^7^	9 × 10^7^	1.6 × 10^8^	7 × 10 ^7^	1.2 × 10^8^	8 × 10^7^	9 × 10^7^	8 × 10^7^	1.5 × 10^8^	5 × 10^7^
10 minutes	3 × 10^8^	8 × 10^7^	5 × 10^7^	4 × 10^7^	1.1 × 10^8^	1.5 × 10^8^	1.3 × 10^8^	1 × 10^8^	7 × 10^7^	5 × 10^7^	6 × 10^7^	6 × 10^7^
30 minutes	2.7 × 10^8^	1 × 10^8^	1.3 × 10^8^	3 × 10^7^	8.4 × 10^7^	1.6 × 10^8^	9 × 10^7^	1.2 × 10^8^	4 × 10^8^	8 × 10^7^	6 × 10^7^	5.3 × 10^7^
60 minutes	2.2 × 10^8^	1.5 × 10^8^	5 × 10^7^	3 × 10^7^	3 × 10^8^	3 × 10^8^	6 × 10^7^	1.5 × 10^8^	1.2 × 10^8^	8 × 10^7^	5 × 10^7^	4.3 × 10^7^
120 minutes	5 × 10^8^	1.3 × 10^8^	1 × 10^8^	2.5 × 10^8^	5 × 10^8^	4 × 10^8^	4 × 10^8^	6 × 10^8^	3 × 10^8^	9 × 10^8^	4 × 10^7^	1 × 10^8^

GC, growth control; GP, genipin.

* ≥1-log reduction.

† ≥3-log reduction.

‡ The positive growth control value against which the log-1 and log-3 reductions are compared.

## Discussion

### MBC and MIC

In this study, genipin demonstrated promising antibacterial activity against pathogens commonly associated with infected ulcerative keratitis. MBC values ranged from 0.075% to 0.125% for the *Staphylococcus* spp. and *P. aeruginosa.* These values align with previously reported MBCs of 0.156% against ATCC 27853 *P. aeruginosa* and the slightly higher 0.321% against ATCC 25923 *S.* *aureus* in an ex vivo porcine model of bacterial keratitis.^26^

The deep blue discoloration of the content in the wells interfered with both visual and spectrophotometric assessment, rendering both visual inspection and OD₆₀₀ measurements inadequate for MIC determination of genipin. Because the discoloration did not occur when the broth was replaced with 0.85% saline solution, this discoloration likely arises from genipin reacting with free amines present in nutrient broths or from interactions associated with bacterial proliferation (saline lacks nutrients to support bacterial growth). Previous reports on the MBC of genipin did not include MIC values nor clarify the reason for their exclusion.^25^^,^^26^ One study did report an MIC of genipin against *E. coli*^27^; however, the methodology did not appear to adhere to CLSI, EUCAST, or other established guidelines, rendering the results questionable. Future work could assess MIC determination using alternative OD wavelengths and evaluate whether differences in nutrient composition alter genipin reaction kinetics.

In our study, MRSP exhibited similar susceptibility to genipin as *S. pseudintermedius*, suggesting that genipin might hold potential against MDR bacterial strains. Considering the high prevalence of MDR bacteria in human and veterinary ophthalmology,^7^^,^^9^^,^^14^^,^^15^ it is worth further investigating genipin's efficacy against drug-resistant isolates.

This study is the first to report the antibacterial activity of genipin against a *Streptococcus* species. Compared with *Staphylococcus* spp. and *P. aeruginosa*, the MBC value for *S. canis* was notably higher under microaerophilic conditions (0.5%) but decreased to 0.1% under aerobic conditions, aligning with the values observed for the other isolates. Notably, many *Streptococcus* strains grow optimally under microaerophilic or CO₂-enriched conditions,^47^ indicating that *S. canis* may exhibit poorer growth under aerobic conditions. Although in vitro findings are not directly translatable to in vivo settings, it is worth noting that the corneal environment is generally aerobic; however, anaerobic conditions develop during eyelid closure.^48^ Furthermore, in case of a corneal injury or inflammation, corneal hypoxia can develop when a higher oxygen requirement exceeds the oxygen supply.^49^ This illustrates the complexity of contributing factors in corneal infections.

### Time-Kill Analysis

Although several drug characteristics (e.g., tissue binding, lipophilicity, molecular size) determine their local efficacy, faster-acting agents are logically preferred for topical application. This is particularly important in ophthalmic formulations, where tear film dynamics can rapidly dilute and clear the drug,^50^ reducing its effective concentration. In this study, 0.25% genipin achieved a mean bactericidal effect against staphylococci within two hours, whereas 0.5% genipin did so within one hour, supporting the use of higher genipin concentrations in corneal infections. Similarly, higher genipin concentrations resulted in deeper corneal crosslinking (CXL) effects.^51^ However, increasing genipin concentrations may raise safety concerns. In vivo studies in rabbits have shown that topical application of 0.2% and 0.25% genipin onto experimentally induced epithelial defects caused minimal to no damage to corneal nerves, keratocytes, and endothelial cells,^39^^,^^40^^,^^51^ and proved safer than UVA-CXL,^39^^,^^51^ whereas 0.3% genipin resulted in significant keratocyte apoptosis and endothelial cell damage.^40^ Furthermore, the cornea exhibited marked structural alterations in an ex vivo study using porcine corneal strips treated with 1% genipin.^33^ This dose‑dependent genipin cytotoxicity potentially arises because low genipin concentrations likely primarily target extracellular matrix proteins, whereas higher concentrations induce more extensive intracellular protein crosslinking and reactive oxygen species–mediated apoptosis.^52^^,^^53^ An additional concern is that genipin could affect tissues surrounding the targeted treatment area, as it acts as a natural crosslinker for any molecule with primary amine groups, including the epithelial layers of the conjunctiva and skin. However, an intact corneal epithelium appears to block the CXL effects of genipin.^30^ Similarly, no blue discoloration was reported when a single dose of 0.34% genipin was administered on an intact rabbit cornea,^26^ whereas a slight blue hue in the treated corneal stroma was reported in several human patients treated with 0.2% genipin-soaked cellulose sponges for five minutes.^29^ Clinical application methods and potential adverse effects need to be further explored.

Although 0.1% genipin proved bactericidal against *P. aeruginosa* after 22 hours of incubation, the bacterial strain was not affected by genipin concentrations up to 0.5% within the 120-minute time-kill assay. Similar limited antibacterial effects against *P. aeruginosa* were observed when 0.34% genipin was applied in two separate doses in experimentally induced keratitis in rabbits.^28^ The restrictive outer membrane of Gram-negative bacteria often limits the permeation of antimicrobial agents, contributing to reduced susceptibility compared to Gram-positives.^54^^,^^55^ Genipin's reactivity with primary amines and protein cross-linking, which may effectively disrupt bacterial cells, could therefore be slower or less efficient in Gram-negative bacteria. Future studies may explore whether incorporating benzalkonium chloride as an additive agent could enhance genipin's penetration and accelerate its antibacterial action.^56^^–^^58^

### Limitations

Like Koudouna et al.,^26^ we did not observe an impact on bacterial growth of DMSO concentrations up to 7.5%. However, consistent with the reported concentration-dependent antibacterial activity of DMSO,^59^^,^^60^ our findings show that a 10% concentration exhibited bacteriostatic activity against *S.* *canis*. Moreover, previous studies have shown that non-growth-inhibitory concentrations of DMSO can alter bacterial gene expression and epigenetic patterns,^61^ suggesting that DMSO may exert indirect antibacterial effects. Additionally, DMSO significantly enhanced the antimicrobial activity of plant essential oils.^62^ Potential contribution to the observed outcomes in our study and previous studies can therefore not be excluded. Nevertheless, these indirect antibacterial effects could prove beneficial from a clinical perspective. Beyond its antibacterial properties, DMSO can penetrate phospholipid bilayers and has recently been proposed as a potential therapeutic agent and drug delivery vehicle, particularly for ocular diseases.^63^

Our study did not account for drug carryover (residual antimicrobial during plating), which may have influenced time-kill assay results. However, genipin showed no effect against *P.* *aeruginosa* within 120 minutes, yet demonstrated apparent bactericidal activity after 22 hours in the MBC assay. This suggests that drug carryover likely had minimal impact on the results.

Additionally, results from in vitro studies are not directly applicable to clinical practice. However, they do provide valuable insights for future study designs.

## Conclusions

The present study supports the potential of genipin as an antibacterial agent for the treatment of infected ulcerative keratitis. However, potential ocular or systemic adverse effects must be thoroughly evaluated before clinical application. For instance, genipin suppresses wound-induced migration/proliferation of subconjunctival fibroblasts and reduces collagen type I, TGFβ1, and αSMA expression.^64^ Additionally, limited in vitro studies show conflicting results regarding its genotoxic potential.^65^^,^^66^ Beyond safety, further research should address genipin's mechanism of action, stability in pharmaceutical formulations, corneal penetration, and pharmacokinetics. In conclusion, genipin exhibited in vitro bactericidal activity against *S. pseudintermedius*, MRSP, *P. aeruginosa*, and *S. canis*, and the time-kill analysis indicated that genipin acts as a concentration-dependent antimicrobial agent.

## Supplementary Material

Supplement 1
