# Supplementary material for: The In Vitro Antibacterial Effects of Genipin Against Pathogens Commonly Associated With Infected Ulcerative Keratitis: A Canine Preliminary Study
Source: Invest Ophthalmol Vis Sci. 2026 Mar 9;67(3):18. doi: 10.1167/iovs.67.3.18 (PMC12988688; doi:10.1167/iovs.67.3.18)
Supplement: Supplement 1 [file iovs-67-3-18_s001.docx]

**TABLES AND FIGURES SUPPLEMENTAL FILES**


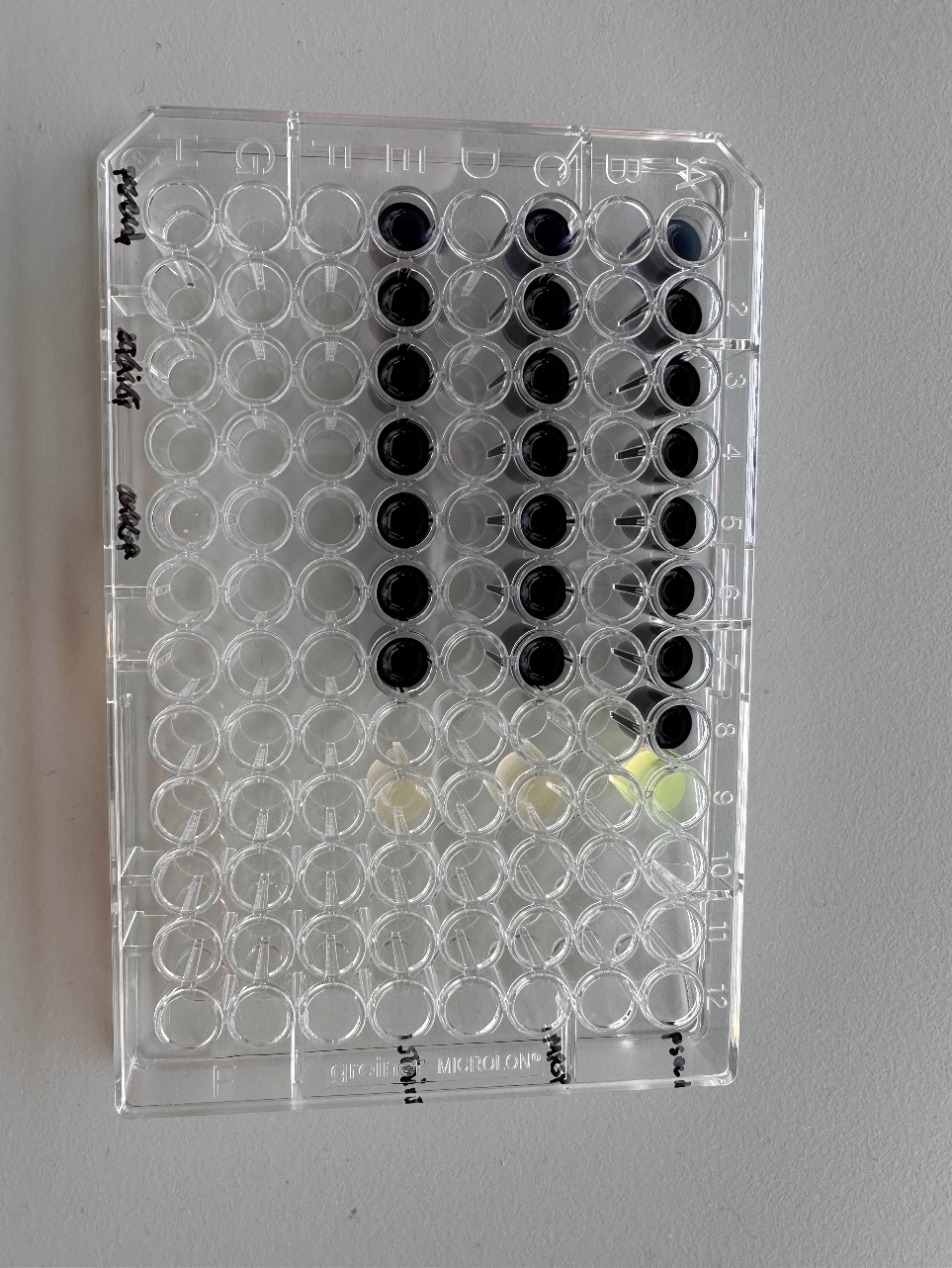


**Supplemental Figure 1.1** Appearance of the 96-well plate after 20 hours of incubation of three bacterial strains in seven different genipin concentrations, including one sterility control and three growth controls. Row A, column 1-7, and 9: *P. aeruginosa*, Row C, column 1-7, and 9: *S. pseudintermedius*, Row E, column 1-7, and 9: Methicillin-resistant *S. pseudintermedius*. Column 1-7, row A, C, and E consisted of the following genipin concentrations: 0.4%, 0.8%, 1.25%, 1.7%, 2%, 2.5%, 3.1%. Column 8: CAMHB and genipin 2% (sterility control). Column 9, row A, C, and E: CAMHB (growth controls). The remaining wells are empty.

| **Before incubation** | | |  |  |  |  |  |  |  |  |  |  |
| --- | --- | --- | --- | --- | --- | --- | --- | --- | --- | --- | --- | --- |
|  |  |  |  |  |  |  |  |  |  |  |  |  |
|  | **1** | **2** | **3** | **4** | **5** | **6** | **7** | **8** | **9** | **10** | **11** | **12** |
| **A** | 0,056 | 0,066 | 0,061 | 0,063 | 0,062 | 0,061 | 0,063 | 0,063 | 0,057 | 0,029 | 0,029 | 0,029 |
| **B** | 0,031 | 0,029 | 0,03 | 0,027 | 0,03 | 0,03 | 0,027 | 0,03 | 0,03 | 0,032 | 0,029 | 0,03 |
| **C** | 0,072 | 0,074 | 0,073 | 0,081 | 0,078 | 0,075 | 0,073 | 0,03 | 0,073 | 0,03 | 0,03 | 0,031 |
| **D** | 0,03 | 0,028 | 0,027 | 0,03 | 0,031 | 0,026 | 0,03 | 0,031 | 0,031 | 0,03 | 0,028 | 0,03 |
| **E** | 0,068 | 0,07 | 0,076 | 0,07 | 0,075 | 0,073 | 0,076 | 0,029 | 0,074 | 0,029 | 0,03 | 0,032 |
| **F** | 0,032 | 0,03 | 0,032 | 0,029 | 0,028 | 0,03 | 0,032 | 0,029 | 0,03 | 0,028 | 0,034 | 0,027 |
| **G** | 0,034 | 0,029 | 0,03 | 0,029 | 0,03 | 0,03 | 0,028 | 0,03 | 0,028 | 0,03 | 0,033 | 0,036 |
| **H** | 0,024 | 0,033 | 0,026 | 0,029 | 0,027 | 0,033 | 0,033 | 0,027 | 0,028 | 0,031 | 0,029 | 0,025 |
|  |  |  |  |  |  |  |  |  |  |  |  |  |
| **After 20 hours of incubation** | | | | |  |  |  |  |  |  |  |  |
|  |  |  |  |  |  |  |  |  |  |  |  |  |
|  | **1** | **2** | **3** | **4** | **5** | **6** | **7** | **8** | **9** | **10** | **11** | **12** |
| **A** | 3,5 | 3,381 | 3,124 | 3,5 | 3,387 | 3,5 | 3,367 | 3,357 | 1,45 | 0,032 | 0,028 | 0,03 |
| **B** | 0,029 | 0,03 | 0,029 | 0,029 | 0,033 | 0,032 | 0,028 | 0,031 | 0,029 | 0,029 | 0,03 | 0,035 |
| **C** | 3,369 | 3,384 | 3,11 | 3,313 | 3,331 | 3,5 | 3,5 | 0,029 | 0,737 | 0,028 | 0,03 | 0,03 |
| **D** | 0,029 | 0,03 | 0,032 | 0,03 | 0,035 | 0,03 | 0,026 | 0,033 | 0,027 | 0,029 | 0,027 | 0,032 |
| **E** | 3,5 | 3,5 | 3,5 | 3,5 | 3,267 | 3,5 | 3,48 | 0,031 | 1,046 | 0,033 | 0,033 | 0,032 |
| **F** | 0,031 | 0,029 | 0,032 | 0,031 | 0,03 | 0,032 | 0,055 | 0,03 | 0,029 | 0,032 | 0,032 | 0,027 |
| **G** | 0,031 | 0,032 | 0,034 | 0,029 | 0,034 | 0,063 | 0,03 | 0,035 | 0,031 | 0,033 | 0,033 | 0,033 |
| **H** | 0,025 | 0,03 | 0,027 | 0,061 | 0,029 | 0,034 | 0,035 | 0,029 | 0,031 | 0,031 | 0,034 | 0,026 |

**Supplemental Figure 1.2** Spectrophotometry results OD 600 of the first genipin pilot assay, prior to and after 20 hours of incubation at 37°C. Absorbance values are displayed as OD. Row A, column 1-7, and 9: *P. aeruginosa*, Row C, column 1-7, and 9: *S. pseudintermedius*, Row E, column 1-7, and 9: Methicillin-resistant *S. pseudintermedius*. Column 1-7, row A, C, and E consisted of the following genipin concentrations: 0.4%, 0.8%, 1.25%, 1.7%, 2%, 2.5%, 3.1%. Row A, column 8: CAMHB and genipin 2% (sterility control). Column 9, row A, C, and E: CAMHB (growth controls). The remainder of the wells were empty. The yellow background in the figure indicates the color of the CAMHB, and the blue background demonstrates the blue discoloration of the content in the wells after 20 hours of incubation.

**
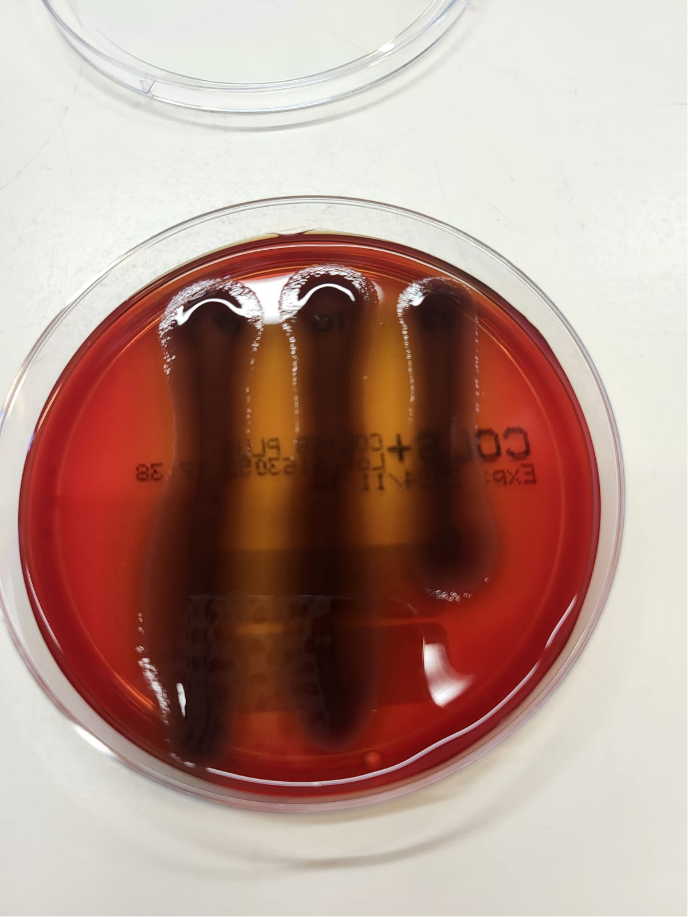

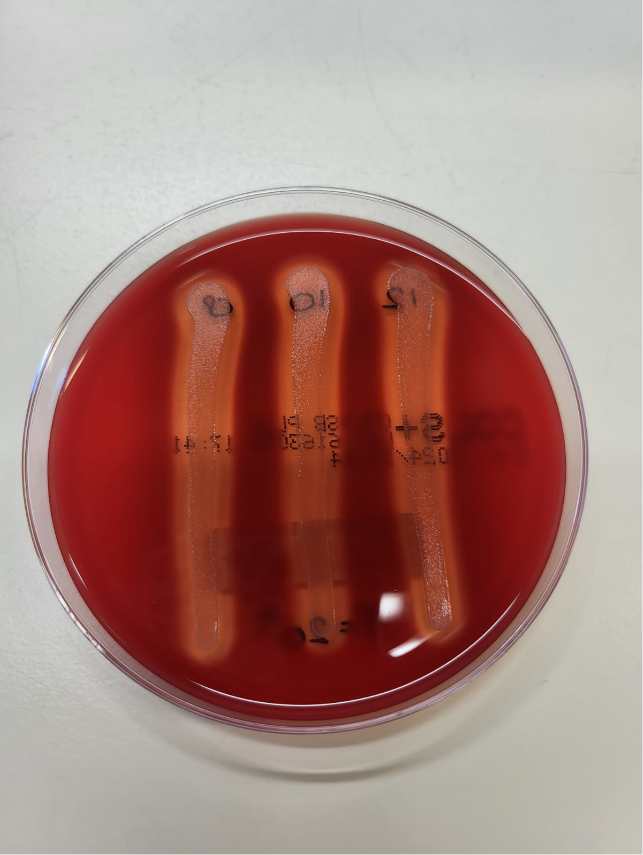

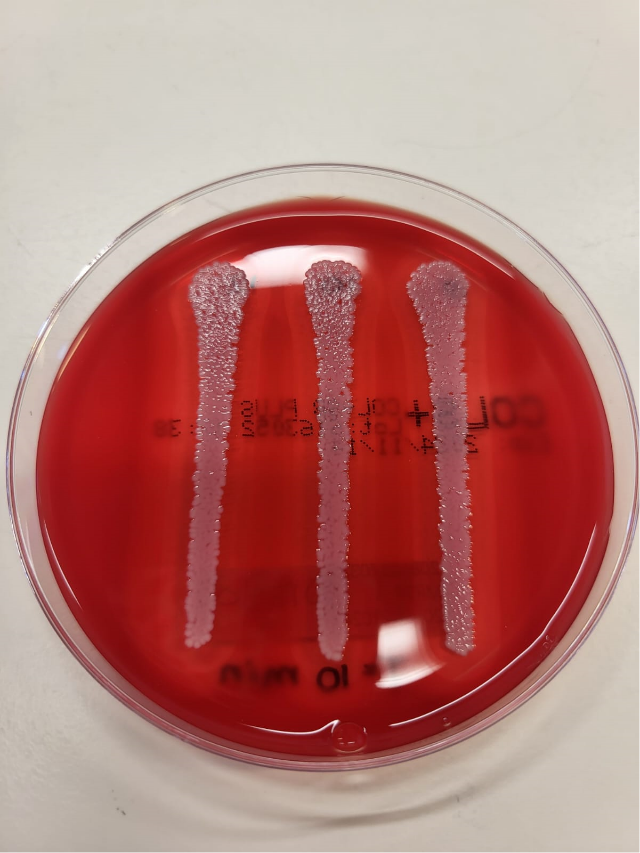

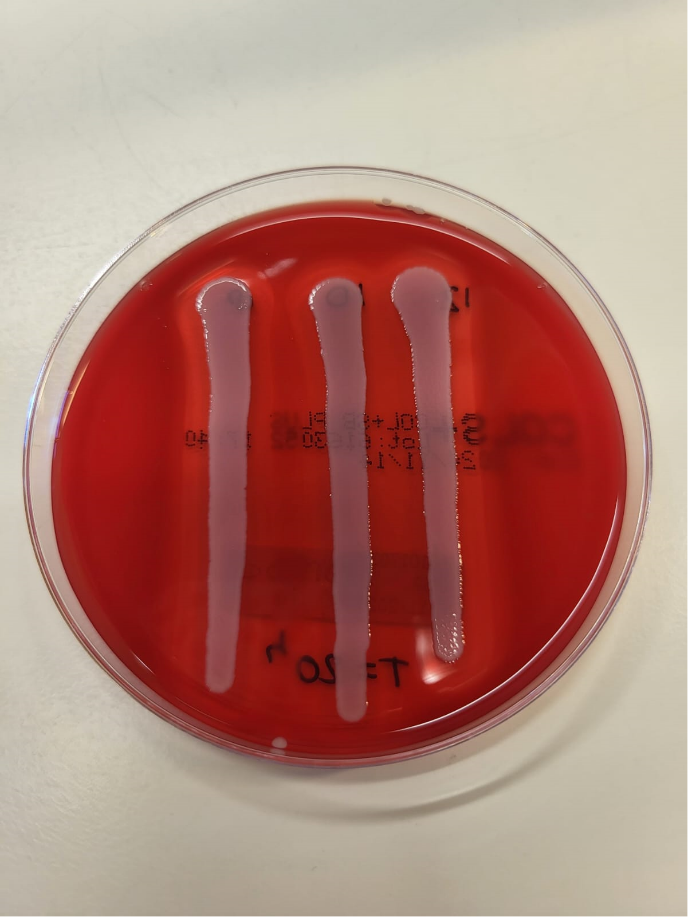
A B**

**C D**


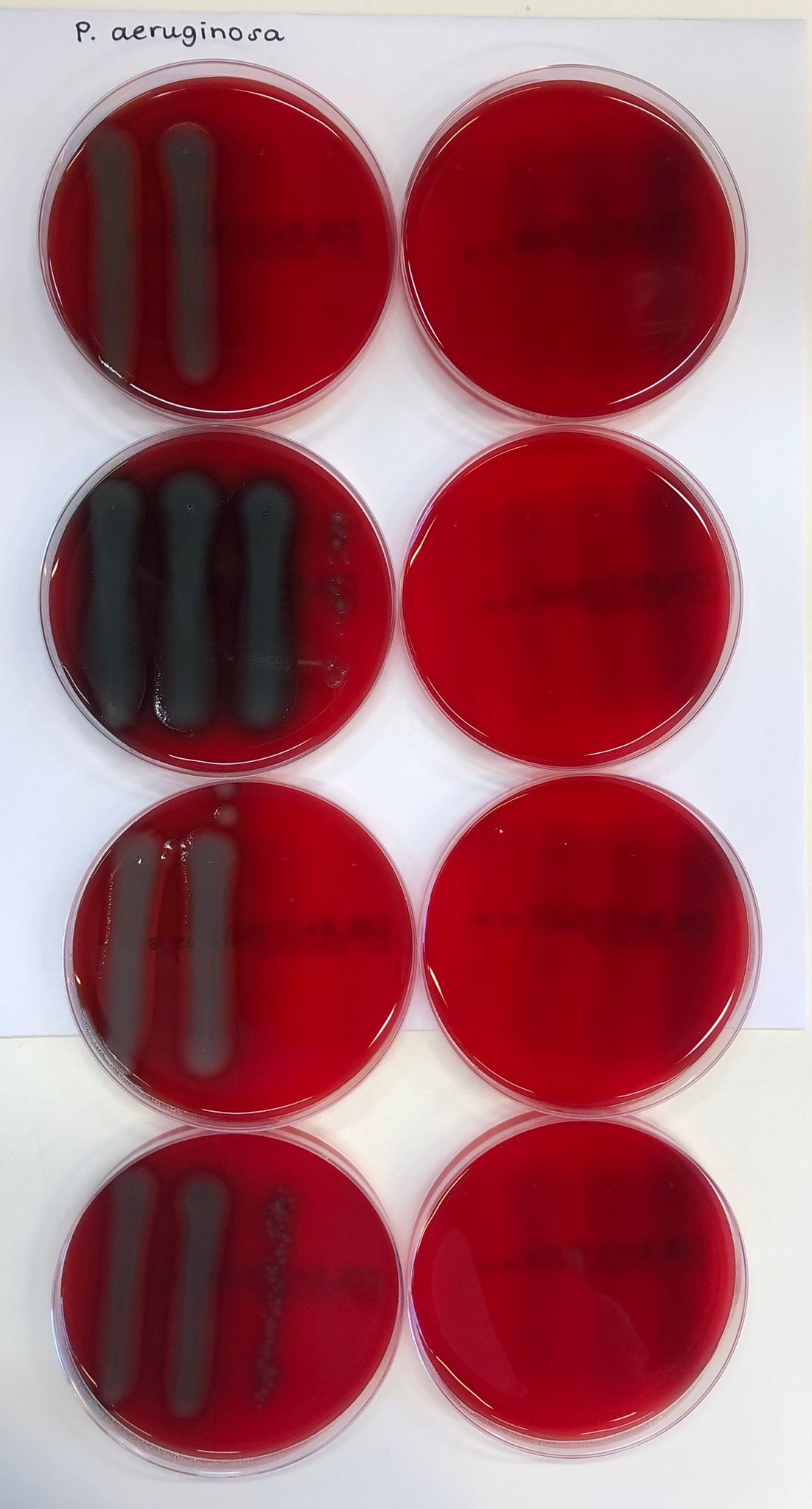

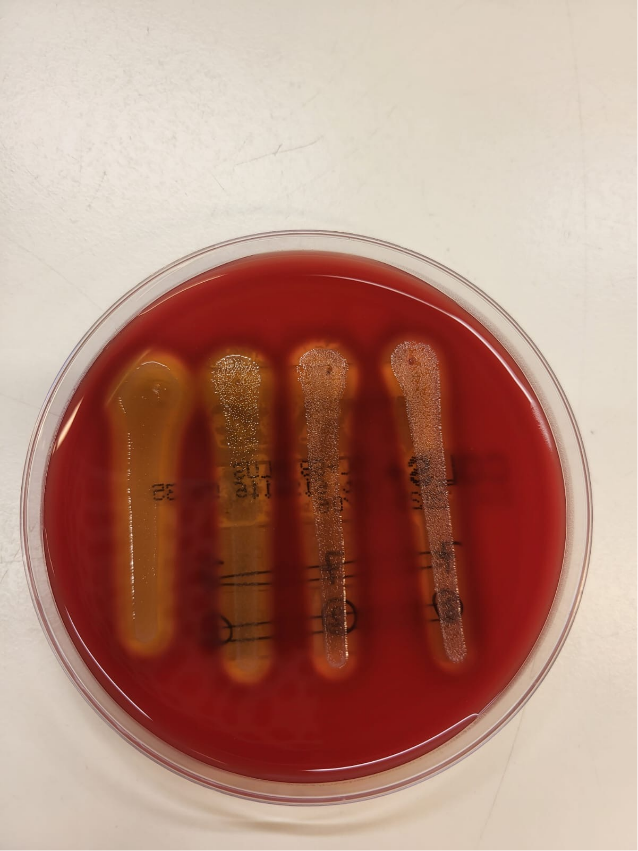


**E F**

**Supplemental Figure 1.3** Tracking dilution on blood agar plates. A-D: 3 tracks, E and F: 4 tracks. A: MRSP, B: *S. pseudintermedius*, C: *S. canis*, D: *P. aeruginosa*. E: *S. canis*, F: *P. aeruginosa*. Note that no bacterial growth is on the track to the far right.


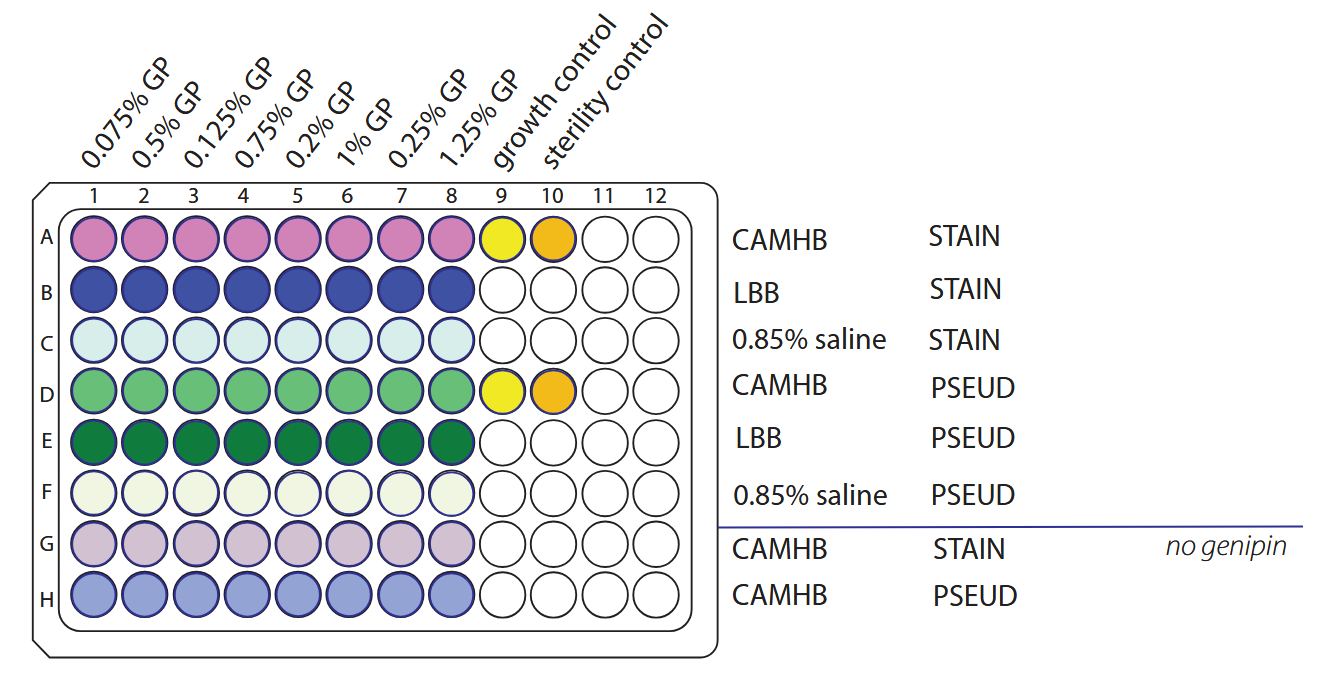


**A**


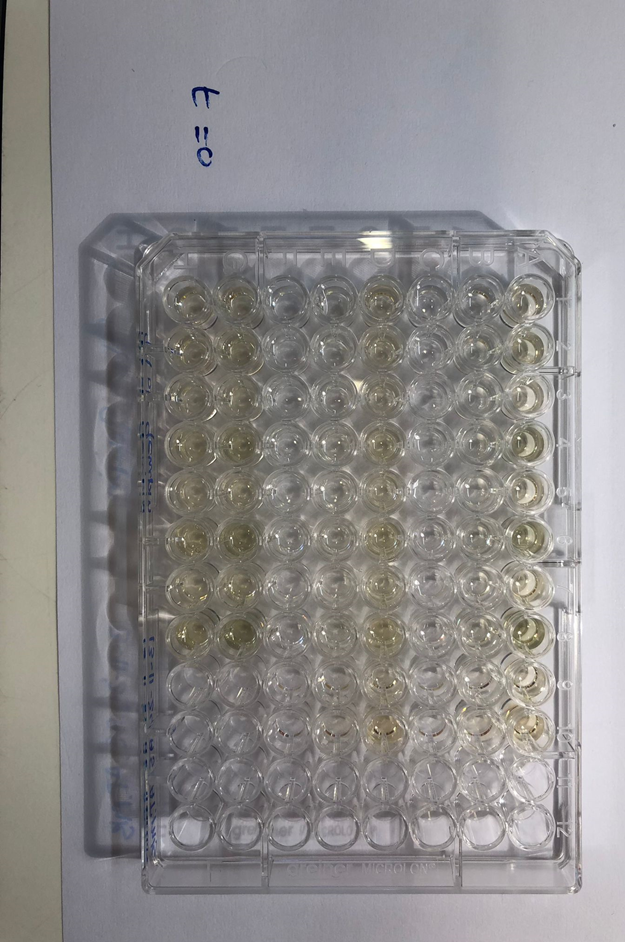


**B**

**
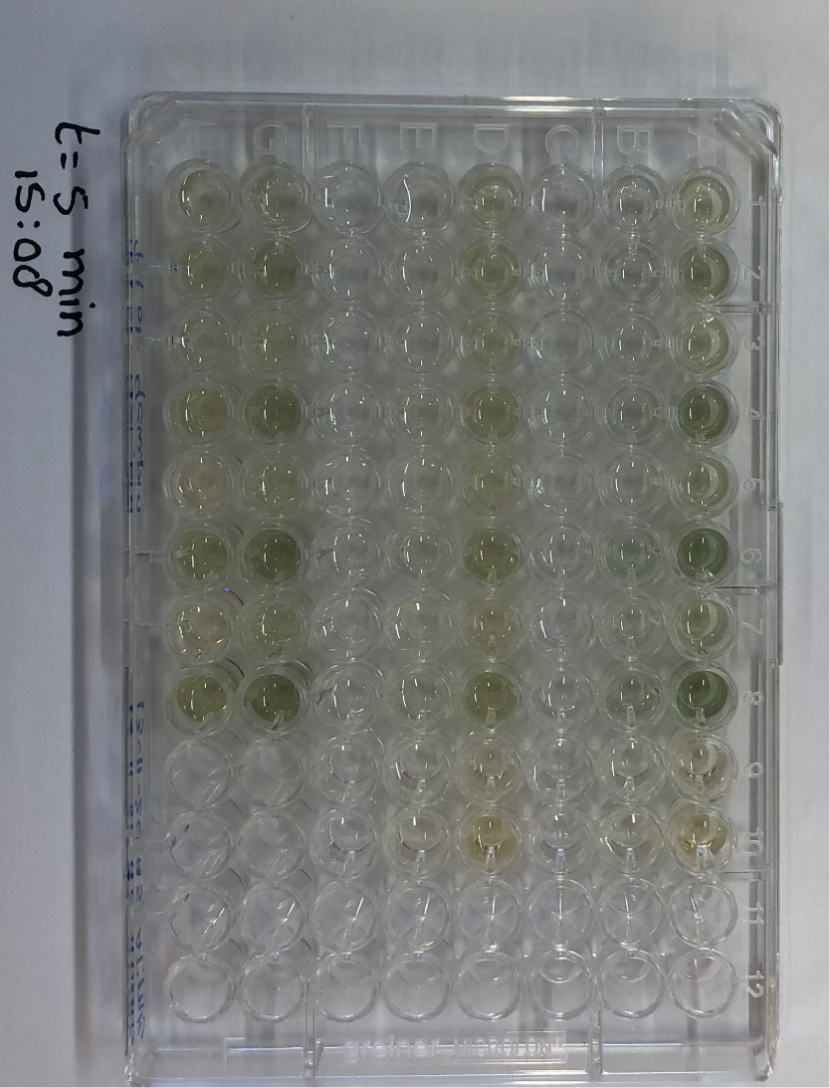
**

**C**

**
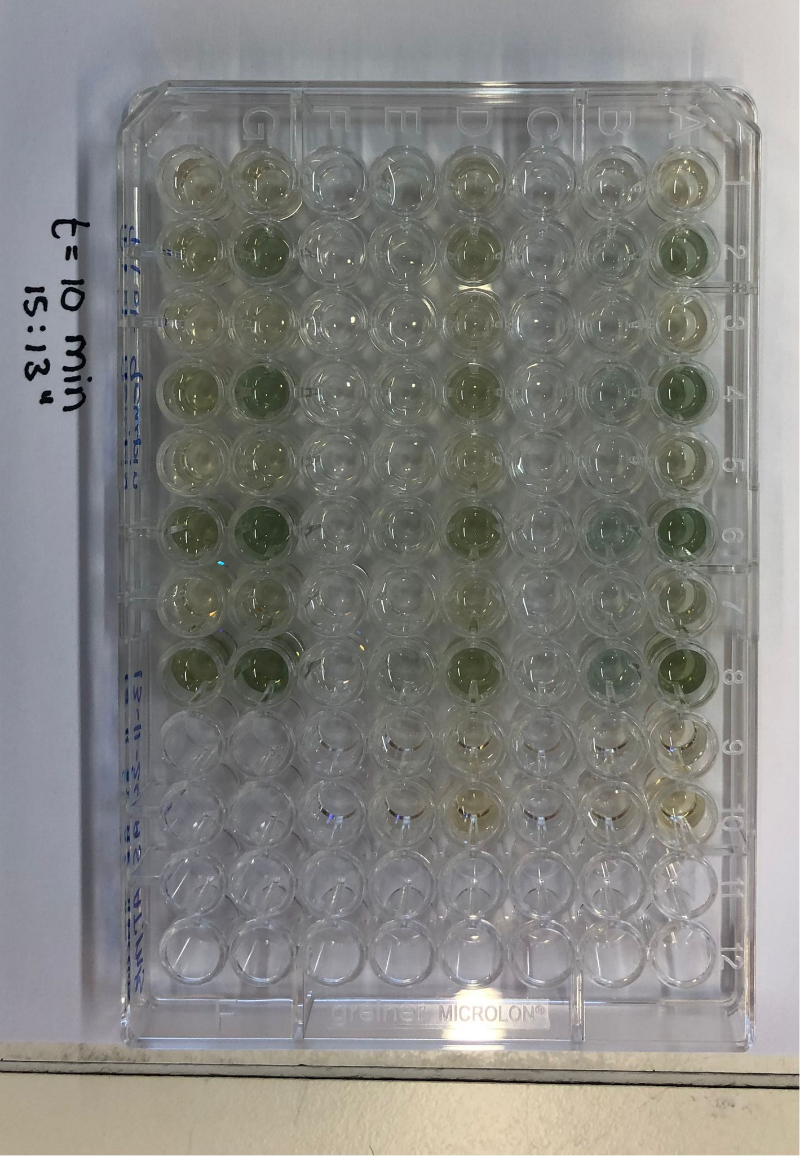
**

**D**


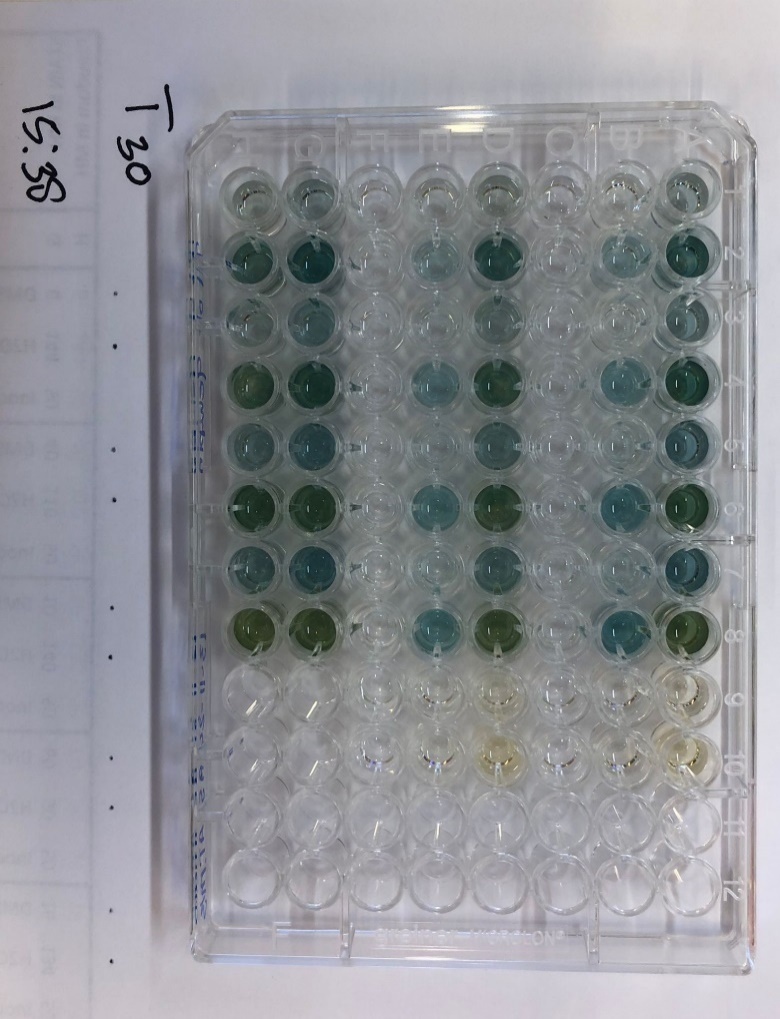


**E**


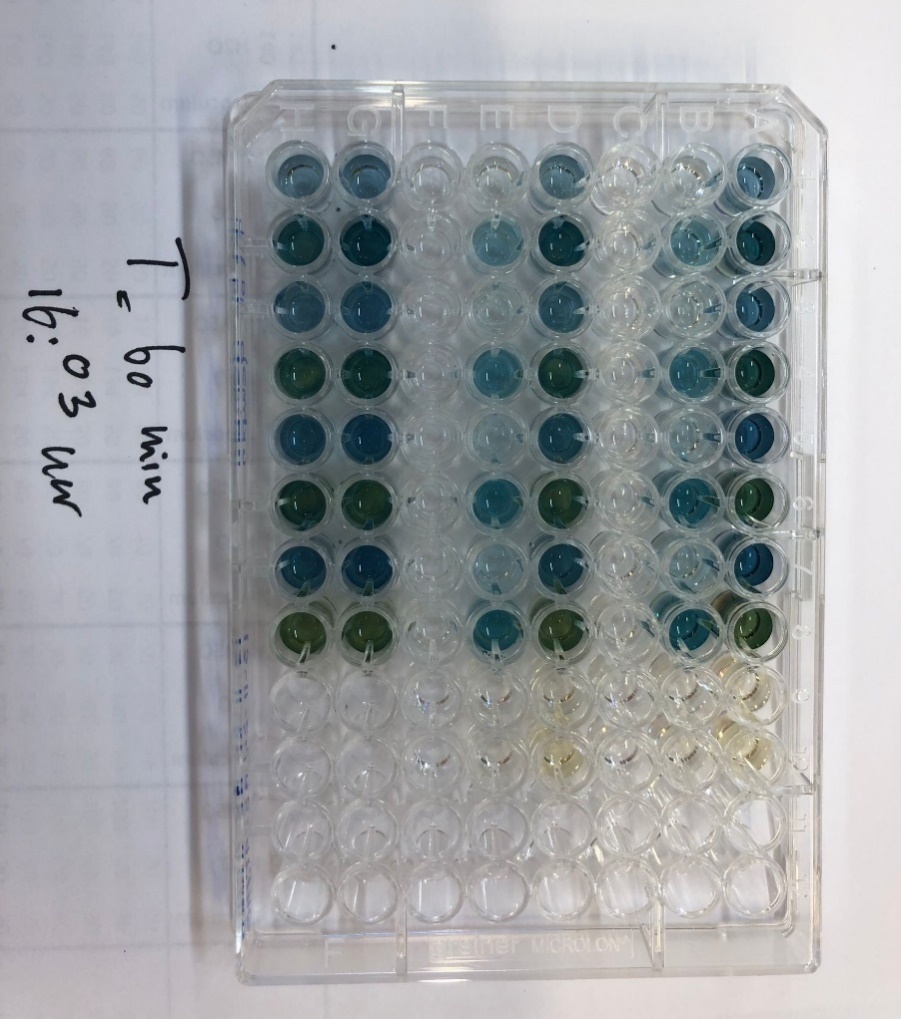


**F**


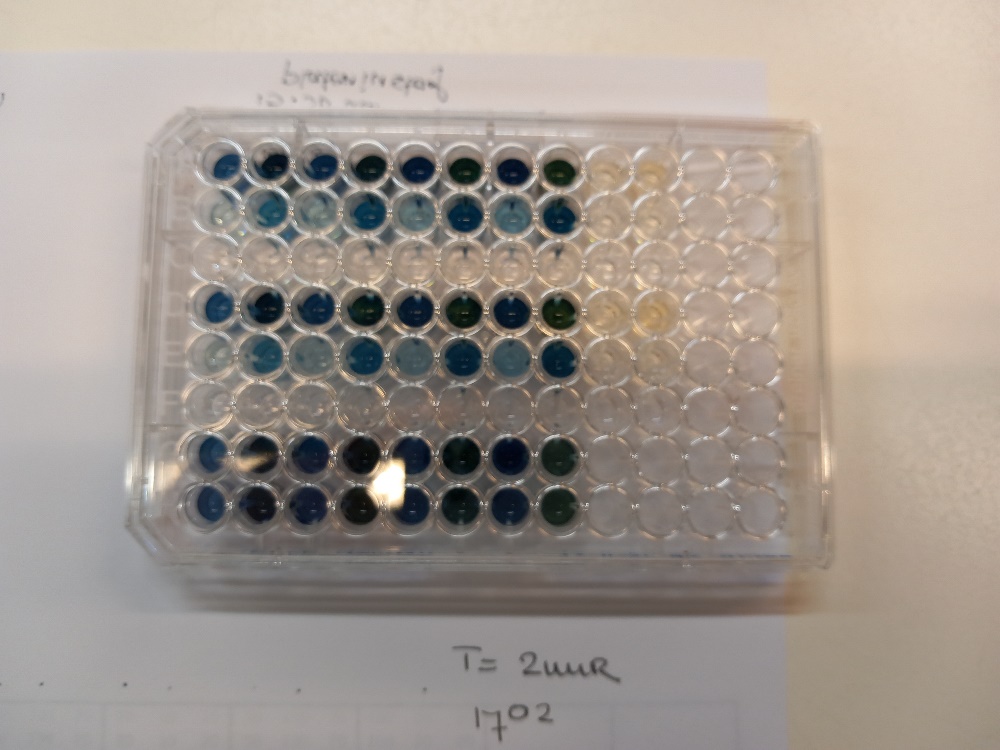


**G**


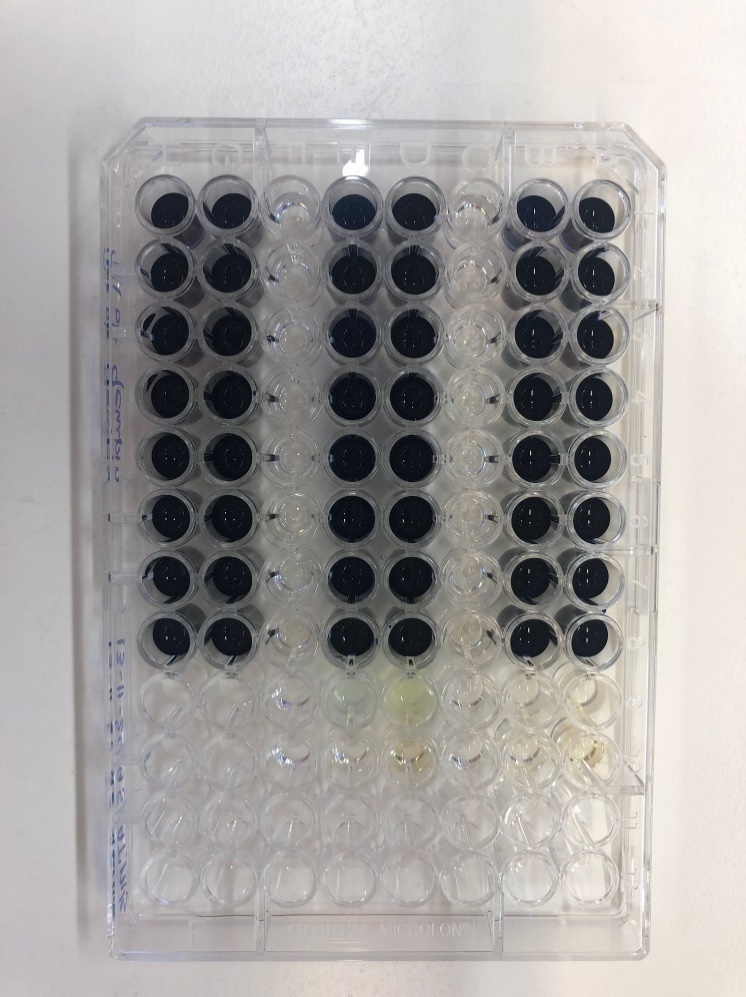


**H**

**Supplemental Figure 1.4** Blue discoloration of the content in the wells per time point. GP = genipin, CAMHB = cation-adjusted Mueller-Hinton broth, LBB = Luria-Bertani broth, STAIN = *Staphylococcus pseudintermedius*, PSEUD = *Pseudomonas aeruginosa*, A: Schematic representation of the plate layout. B. Plate directly after dispensing. C. Plate after 5 minutes of incubation. D. Plate after 10 minutes of incubation. E. Plate after 30 minutes of incubation. F. Plate after 60 minutes of incubation. G. Plate after 120 minutes of incubation. H. Plate after 20 hours of incubation. Note: incubation was performed aerobically at 37°C. Note that the genipin concentrations are not increasing per column (Figure 1.3.A). Genipin concentrations were dispensed alternately to facilitate the tracking dilution assay assessment. Also note that the bottom two rows were assigned to *S. pseudintermedius* and *P. aeruginosa* in CAMHB against varying concentrations of DMSO without genipin. These wells were mistakenly filled with genipin concentrations instead of DMSO; hence, the blue discoloration is also present.

| Bacterial isolates | Internal reference number | Agar | Environment | MBC |
| --- | --- | --- | --- | --- |
| *S. pseudintermedius* | 2110209036 | Blood agar | aerobic | 0.125% |
| *S. pseudintermedius* | 2110209036 | LB agar | aerobic | 0.075% |
| MRSP | 2130426050 | Blood agar | aerobic | 0.15% |
| *P. aeruginosa* | 113420 | Blood agar | aerobic | 0.125% |
| *P. aeruginosa* | 113420 | LB agar | aerobic | 0.125% |
| *S. canis* | 221111806401-1 | Blood agar | microaerophilic | 0.5% |
| *S. canis* | 221111806401-1 | Blood agar | aerobic | 0.1% |

**Supplemental Table 1.1** Preliminary minimum bacterial concentration (MBC) results for genipin against four clinical strains of bacteria commonly associated with canine infected ulcerative keratitis. LB = Luria Bertani. S. *pseudintermedius* = *Staphylococcus pseudintermedius*. MRPS = Methicillin-resistant *Staphylococcus pseudintermedius. P. aeruginosa* = *Pseudomonas aeruginosa. S. canis* = *Streptococcus canis.*

| Bacterial isolates | Internal reference number | Agar | Environment | DMSO | Bacterial growth | CFU/ml | Growth control |
| --- | --- | --- | --- | --- | --- | --- | --- |
| *S. pseudintermedius* | 2110209036 | Blood agar | aerobic | 2 % | + | - | + |
| *S. pseudintermedius* | 2110209036 | Blood agar | aerobic | 2.5 % | + | - | + |
| *S. pseudintermedius* | 2110209036 | Blood agar | aerobic | 5 % | + | - | + |
| MRSP | 2130426050 | Blood agar | aerobic | 2 % | + | - | + |
| MRSP | 2130426050 | Blood agar | aerobic | 2.5 % | + | - | + |
| MRSP | 2130426050 | Blood agar | aerobic | 5 % | + | - | + |
| *P. aeruginosa* | 113420 | Blood agar | aerobic | 2 % | + | - | + |
| *P. aeruginosa* | 113420 | Blood agar | aerobic | 2.5 % | + | - | + |
| *P. aeruginosa* | 113420 | Blood agar | aerobic | 5 % | + | - | + |
| *S. canis* | 221111806401-1 | Blood agar | microaerophilic | 2 % | + | - | + |
| *S. canis* | 221111806401-1 | Blood agar | microaerophilic | 2.5 % | + | - | + |
| *S. canis* | 221111806401-1 | Blood agar | microaerophilic | 5 % | + | - | + |
| *S. canis* | 221111806401-1 | Blood agar | microaerophilic | 7.5% | +/- | 1.5 x 10^7^ | 3 x 10^7^ |
| *S. canis* | 221111806401-1 | Blood agar | microaerophilic | 10% | < | 1 x 10^6^* | 3 x 10^7^ |

**Supplemental Table 1.2** The antibacterial effects of DMSO. Viability counts (CFU/ml) were performed only when visual assessment of growth and/or subculturing of blood agar plates indicated inhibition of bacterial growth (i.e., the wells were less turbid than the positive growth control, or the tracking dilution showed fewer bacterial colonies). The plus sign indicates positive bacterial growth on visual inspection and confluent bacterial growth (>300 colonies) on blood agar plate by tracking dilution. The plus/minus sign indicates slightly altered turbidity in the well and reduced bacterial growth on blood agar plates. The “less than” sign indicates clear decrease in turbidity and reduced bacterial growth. Asterix indicates a 1-log reduction in bacterial growth.
